# Creatureness

**DOI:** 10.4102/ajod.v3i1.127

**Published:** 2014-11-25

**Authors:** Rosamund (Mindy) Stanford

**Affiliations:** 1 Independent writer and editor

*Left Over* (2013) is Kobus Moolman’s fourth collection of poetry. Knowing the strength of his earlier work I entered this book tentatively, afraid of losing my balance. Afraid of falling over emotionally. My fears were realised. I did not know anymore which was inside and which was outside.

The words are stark and sparse. Ordinary words, ordinary phrases a child would use. In the opening poem ‘Back to school’, the air is ‘hot’ and ‘still’, the next door children are splashing with abandon: they have a swimming pool. A few lines on, ‘he remembers that his parents could only afford to buy the pack of six crayons – that did not have silver or gold or flesh’. The word ‘flesh’ stands out: a child hankering for novelty, an innocent longing (nevermind the presumptuousness of the manufacturer’s calling pale pink ‘flesh’).

Abruptly things slow to a standstill: ‘He stares at the table’. Space looms between the lines. Mastery of the reading pace is a feature of the book as a whole. In this second poem, the words are again ordinary, and the things are ordinary in ordinary relationship: ‘His elbows on the table/His book on the table/…/The light from the overhead lamp flat across the table’. Then, slowly at first, the relationship between things shifts, the boundaries start to melt. Among others, the line between animate and inanimate is obscured: ‘The table makes the humming sound of a refrigerator./The table with its dark underbelly./Its secret place where legs go when they go underneath a table’. The viewer’s seeing of the table becomes an element of the table: ‘The table made out of weight and sight and ten scratches’. ‘Weight’ and ‘sight’ listed along with the seeable and countable ‘ten scratches’, jolts the reader back to the concrete, back to earth – but not quite to the same earth.

In later poems the line between the internal physical body and what is outside becomes permeable. And the sky is a tangible presence: ‘He wonders how much longer/he will be able to hold himself upright/against the sky’ (‘There is something’, p. 16). The more-than-oneness of some elements (such as the sky) is noticeable. Apart from being a prop or holder, the sky is a mirror of incompleteness: ‘There is something missing from the sky. Something his eyes should have seen. But did not have a name for’ (‘Blood x 3 (1)’ p. 30). In ‘Sitting again’ (p. 26), as the world begins to congeal, the sky becomes implacable: ‘Air like a net without any holes in it’.

The menace of the everyday in ‘He looks in the mirror’ (p. 13) is shocking: ‘And suddenly he thinks/how easy it would be/for the chair behind his desk/to plunge a sharpened slat into his back’. The fear is unrelenting, but there is a note of respite: ‘They come again, the dark birds of clamour.//… Only the small chime of silver bells can hold off the clamour’.

We meet hands and feet in unexpected places (and sometimes in unexpected numbers). In ‘And it seems to him’ (p. 12), the rain is ‘beating the world with/small silver hands and feet/beating it into the shape/of something that can be given a name’. Then further on ‘with three hands/he beats back the distractedness/in his heart’. In ‘A warm wind’ (p. 19), we get ‘the smell of sleepless white walls and passages with hands’. Or ‘he cannot understand his hands. They are just two loose things at the ends of his arms. He flaps them. They are heavy. He bites them. And they are hard’ (p. 41). Hands in particular play multiple roles: sometimes agents of action and, as in the last example, near lifeless appendages.

The idea of disembodiment goes a step further into dismemberment. In ‘He cannot understand his hands’, the ‘he’ of the poem follows a macabre line of thought: would chopping off his heavy and unfeeling hands liberate them? ‘Would they bury themselves instantly into the ground like moles?’(p. 41) The ‘creatureness’ of being in a physical body, human or otherwise, is strong in this poem and in some others too. Sometimes it is the inability to feel that is evoked through a creature: ‘The creature has no heart either. Just a vacuum-pump that it manipulates with its huge hands’ (p. 21).

The ‘he’ is a presence observed by the poet with clinical precision. It is ‘he’ whose inside and outside swap places or merge, whose experience of his own anatomy and of the physical world becomes transmogrified. So much so that ‘he’ reminds himself:

That there was not just a front and a back to himself, but, more importantly, an outside. That he was standing up inside a sack of skin that went with him wherever he went, and this was what the rest of the house saw. (‘In the bathroom’, p. 50)

But it is not only the poet (and the reader) and ‘the rest of the house’ observing (or scrutinising) the ‘he’. There is another observer: ‘something was always there with him, watching and listening, through the keyholes of his skin’.

The struggle to hold on takes different forms. With the ‘three hands’ mentioned already, ‘he’ tries to haul himself back to some kind of faith: ‘He looks for a sturdy handhold,/one that will not come away from the wall/if he grabs it with his whole life//and pulls./Pulls himself up/out of his unfaithfulness’. The poem, ‘Hold just like that’, describing a photograph of the partially naked subject is chilling in its helplessness: ‘And the viewer is left with the question: what is the man going to do with the belt?’ (p. 42). Then there is defiance: ‘An iron chain rattles/behind every sentence he stutters.//He steps out into the sun/and strips off all his clothes.// Now God can see exactly/what kind of thing He’s dealing with’ (‘An open vice stands’, p. 53).

The voice is consistent and the pared-down style has the confidence of a highly skilled writer, but the strongest feature is the absence of sentimentality: nobody is telling us what to feel. The poems are bleak and cleanly expressed. They are transformative in the way that *butoh* (the Japanese dance form that emerged after Hiroshima) is: they refuse to be trammeled by emotive or prettifying forms. They exist in an unidealised present: ‘An aloneness that was preparing itself for something. A quiet that had been separated from the rest of the house’ (‘In the bathroom’, p. 50). It is a privilege to read a book so seasoned in form and so naked in content.

